# Seasonal epidemiological and clinical characteristics of pediatric patients with human parainfluenza virus infection by serotype: a retrospective study

**DOI:** 10.1186/s12985-022-01875-2

**Published:** 2022-09-06

**Authors:** Ji Yoon Han, Woosuck Suh, Seung Beom Han

**Affiliations:** 1 Department of Pediatrics, Daejeon St. Mary’s Hospital, The Catholic University of Korea, Daejeon, Republic of Korea; 2 Department of Pediatrics, College of Medicine, The Catholic University of Korea, Seoul, Republic of Korea; 3 Department of Pediatrics, Hangang Sacred Heart Hospital, College of Medicine, Hallym University, 12 Beodeunaru-ro 7-gil, Yeongdeungpo-gu, Seoul, 07247 Republic of Korea

**Keywords:** Parainfluenza virus infections, Seasonal variation, Child, Korea

## Abstract

**Background:**

The development of the polymerase chain reaction (PCR) test promoted the evaluation of the epidemiological and clinical characteristics of human parainfluenza virus (HPIV) type 4, which has been rarely studied using conventional diagnostic methods. This study aimed to determine the seasonal epidemiological and clinical characteristics of all four HPIV serotypes (HPIV-1, HPIV-2, HPIV-3, and HPIV-4) during the era of PCR testing.

**Methods:**

The medical records of hospitalized pediatric patients diagnosed with HPIV infections by a multiplex PCR test between 2015 and 2021 were retrospectively reviewed to determine the seasonal distributions of each HPIV serotype. For patients with a single HPIV infection, the clinical characteristics of each HPIV serotype were evaluated and compared with one another.

**Results:**

Among the 514 cases of HPIV infection, HPIV-1, HPIV-2, HPIV-3, and HPIV-4 were identified in 27.2%, 11.9%, 42.6%, and 18.3% of cases, respectively. HPIV-3 was most prevalent in spring, and the other three serotypes were most prevalent in autumn. For patients with a single HPIV infection, those infected by HPIV-1 and HPIV-3 were younger than those infected by HPIV-2 and HPIV-4 (*P* < 0.001). Croup and lower respiratory tract infection (LRI) were most frequently diagnosed in patients infected by HPIV-1 (*P* < 0.001) and HPIV-4 (*P* = 0.002), respectively. During 2020–2021, HPIV-3 was most prevalent in autumn and caused fewer LRIs (*P* = 0.009) and more seizures (*P* < 0.001) than during 2015–2019.

**Conclusions:**

Each HPIV serotype exhibited a distinct seasonal predominance, and some differences in the clinical characteristics of the HPIV serotypes were observed. HPIV-4 acted as an important cause of LRI. Considering the recent changes in the epidemiological and clinical characteristics of HPIV-3, more time-series analyses should be conducted.

## Background

Human parainfluenza virus (HPIV) is one of the major viral causes of upper respiratory tract infection (URI) and lower respiratory tract infection (LRI) in children, accounting for about 10% of hospitalized children with respiratory tract infections [1, 2]. Also, HPIV is the most common cause of croup, accounting for 38–64% of cases [1–3]. HPIV is an enveloped single-strand, negative-sense RNA virus that belongs to the *Paramyxoviridae* family [4]. HPIV is divided into four genetically and antigenically distinct serotypes (HPIV-1, HPIV-2, HPIV-3, and HPIV-4), and there are two genera of HPIV: *Respirovirus* (HPIV-1 and HPIV-3) and *Rubulovirus* (HPIV-2 and HPIV-4) [4]. Moreover, there are genetically distinct strains in each serotype: three genetic clusters each in HPIV-1 and HPIV-3 and four genetic clusters each in HPIV-2 and HPIV-4 have been reported [4–7]. Because genetic information about HPIV is still insufficient, studies of genetic variation based on the whole genome or hemagglutinin-neuraminidase (HN) gene in each HPIV serotype have been conducted [5–7]. Each HPIV serotype exhibits different epidemiological patterns. Among the four serotypes, infection with HPIV-3 exhibits the highest prevalence, and it peaks in spring and summer [1, 3, 8–11]. Epidemics of HPIV-1 and HPIV-2 infections generally occur in biennial autumn [1, 3, 8–11]. Because of difficulties in isolating the virus, HPIV-4 has rarely been tested using antigen detection tests and cell cultures [4], and resultantly, the seasonality and clinical characteristics of HPIV-4 infection are less defined than those of the other serotypes. The epidemiological and clinical characteristics corresponding to the genetic variations of each HPIV serotype have been poorly studied even for HPIV-3 [12]. With an improved diagnostic sensitivity for HPIV-4 after the introduction of molecular diagnostic tests, such as the polymerase chain reaction (PCR) test [13], various multiplex PCR tests simultaneously identifying the four HPIV serotypes have been recently used in a real-life clinical setting [4]. Although reports on the epidemiological and clinical characteristics of HPIV-4 infection are increasing from the use of PCR tests, various and inconsistent results were reported because of differences in the study design, study duration, geographic area, patient number, and subject selection criteria (e.g., age, diagnosis, and hospitalization status) between the studies [14–19]. Therefore, more studies including various study populations and study years should be performed in each geographic area. In this study, the epidemiological and clinical characteristics of HPIV infection during a 7-year period were determined and compared among the four HPIV serotypes in Korean pediatric patients.

## Methods

### Subjects and study design

Pediatric patients (< 19 years of age) admitted to the Department of Pediatrics at Daejeon St. Mary’s Hospital (Daejeon, Republic of Korea) between January 2015 and December 2021 and underwent evaluations for respiratory viruses using a multiplex PCR test were recruited for this study. Among them, the patients who were positive for HPIV infections were included in this study. Patients who were positive for two or more HPIV serotypes simultaneously were excluded from the study analysis because they could have combined clinical manifestations of the different serotypes. If the same HPIV serotype was identified within 4 weeks after a previous HPIV infection, that episode was excluded, considering prolonged shedding of HPIV unrelated to current symptoms. The electronic medical records of the included patients were retrospectively reviewed to collect the demographic data, including sex and age; clinical data, including the presenting symptoms, diagnosis on discharge, breath sounds, and severe complications (receiving oxygen therapy or intensive care); laboratory data, including the complete blood count, erythrocyte sedimentation rate, and C-reactive protein, aspartate transaminase, alanine transaminase, and lactate dehydrogenase levels; and chest x-ray findings. For the diagnosis on discharge, URI was diagnosed when the patient complained of any respiratory symptoms without abnormal breath sounds and chest x-ray findings. Croup was diagnosed when the patient presented with barking cough and hoarseness with or without stridor. LRI was diagnosed when the patient complained of any respiratory symptoms, which were accompanied by abnormal breath sounds or abnormal chest x-ray findings. Among the LRIs, pneumonia was diagnosed when the chest x-ray abnormality was segmental or lobar consolidation; otherwise, bronchitis/bronchiolitis was diagnosed. If a patient experienced LRI following croup, each episode of croup and LRI was assigned separately. Febrile patients without any respiratory symptoms, abnormal physical examination, and abnormal chest x-ray findings were diagnosed with fever without localizing signs (FWLS). The month, season, and year of the diagnosis of HPIV infection were investigated. Because Korea is located in a northern temperate area, four seasons were defined as follows: spring, March to May; summer, June to August; autumn, September to November; and winter, December to February. The included patients were divided into four groups according to the identified HPIV serotype: the HPIV-1, HPIV-2, HPIV-3, and HPIV-4 groups. For the entire study population, including the patients with a single HPIV infection and those with a co-infection of HPIV and other respiratory viruses, the monthly and seasonal distributions of each HPIV serotype were determined. The clinical characteristics of HPIV infection were determined and compared among the four patient groups in patients with a single HPIV infection only, considering confounding effects of other co-identified viruses. The present study protocol was reviewed and approved by the Institutional Review Board of Daejeon St. Mary’s Hospital, which waived the need for informed consent (approval No. DC22RASI0020).

### Microbiological analysis

Multiplex PCR tests for respiratory viruses were performed on nasopharyngeal swabs using a commercially available Allplex Respiratory Panel 1/2/3 kit (Seegene Inc., Seoul, Republic of Korea). The Allplex Respiratory Panel 1/2/3 kit is a one-step real-time RT-PCR assay based on multiple detection temperature technology [20], and simultaneously identifies 16 respiratory viruses in a single fluorescence channel without melting curve analysis: HPIV (types 1–4), adenovirus, bocavirus, coronavirus (229E, NL63, and OC43), enterovirus, human metapneumovirus, influenza A and B viruses with subtyping, respiratory syncytial virus (RSV; groups A and B), and rhinovirus. Nucleic acid extraction was performed using a GeneAll® Viral DNA/RNA Extraction kit (GeneAll Biotechnology Co., Seoul, Republic of Korea) according to the manufacturer`s instruction. Real-time RT-PCR was performed using a CFX96 real-time PCR detection system (Bio-Rad, Hercules, CA, USA). Automated analysis of the results was performed using a Seegene Viewer software (Seegene Inc.). Samples were considered positive when the cycle threshold was < 42.

### Statistical analysis

Categorical data were compared using a chi-square test between the patient groups, and continuous data were compared using the Mann–Whitney test (between two groups) or Kruskal–Wallis test (between four groups). The proportions of each serotype according to the month and season were compared using the chi-square test. The SPSS 21 software program (IBM Corporation, Armonk, NY, USA) was used to perform all of the statistical analyses. The threshold of statistical significance was defined as a *P*-value of 0.05.

## Results

Between January 2015 and December 2021, a total of 4517 multiplex PCR tests were performed in pediatric inpatients, and 3298 (73.0%) of them were positive for at least one respiratory virus. HPIV was identified in 521 (11.5%) multiplex PCR tests, and four episodes in which two HPIV serotypes were identified concurrently were excluded. Three patients were repeatedly positive for the same HPIV serotype within 4 weeks after a previous infection. They had concurrent rhinovirus or bocavirus infection on the second test, and their second episodes of HPIV infection were excluded. For the remaining 514 episodes of HPIV infection (302 [58.8%] males and 212 [41.2%] females), the median age of the included patients were 22 months (interquartile range 13–36), and 384 (74.7%) of them were aged < 36 months. HPIV was co-identified with other respiratory viruses in 324 (63.0%) episodes and identified singly in 190 (37.0%) episodes. HPIV-1, HPIV-2, HPIV-3, and HPIV-4 infections were identified in 140 (27.2%), 61 (11.9%), 219 (42.6%), and 94 (18.3%) episodes, respectively. The co-identification rates of HPIV-1, HPIV-2, HPIV-3, and HPIV-4 with other viruses were 65.0% (*n* = 91), 70.5% (*n* = 43), 61.6% (*n* = 135), and 58.5% (*n* = 55), respectively (*P* = 0.441).

### Epidemiology of HPIV infection by serotype

**Fig. 1:**
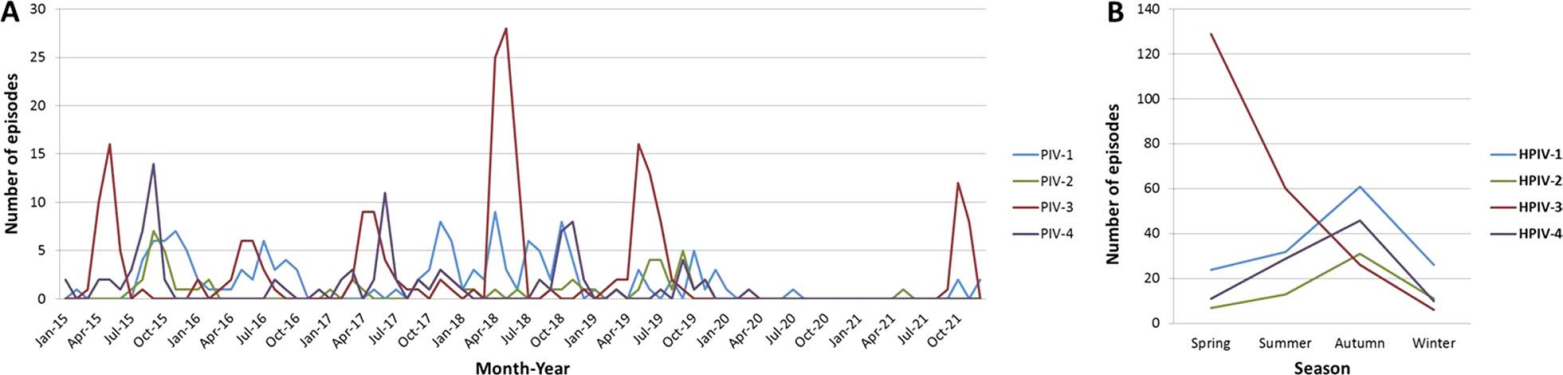
Monthly **A** and seasonal **B** distributions of the identified HPIV by serotype. *HPIV* Human parainfluenza virus

The monthly and seasonal distributions of each HPIV serotype were determined for the 514 episodes of HPIV infection (Fig. 1). Because of the pandemic of coronavirus disease 2019 (COVID-19), only 3 and 26 episodes were identified in 2020 and 2021, respectively. Before the COVID-19 pandemic in 2015–2019, HPIV-3 was the most prevalent of four serotypes every year, with annual peaks between April and June (Fig. 1A). HPIV-1 showed a less-prominent seasonal peak than HPIV-3, and HPIV-2 and HPIV-4 showed various seasonal patterns in each year (Fig. 1A). However, over the study period, HPIV-1, HPIV-2, and HPIV-4 showed seasonal peaks in autumn (Fig. 1B). During the COVID-19 pandemic in 2021, HPIV-3 showed a seasonal peak in autumn, which was different from previous epidemical patterns for this serotype (Fig. 1A).

### Clinical characteristics of HPIV infection by serotype

The clinical characteristics of HPIV infection were evaluated in 190 patients with a single HPIV infection, and they were compared among the HPIV-1, HPIV-2, HPIV-3, and HPIV-4 groups (Table 1). The patients in the HPIV-1 and HPIV-3 groups were younger than those in the HPIV-2 and HPIV-4 groups (*P* < 0.001). Most patients in the HPIV-1 and HPIV-3 groups were aged < 36 months, while about half of the patients in the HPIV-2 and HPIV-4 groups were aged ≥ 36 months (Table 1). Fever developed least frequently (*P* < 0.001) and fever duration was shortest (*P* = 0.005) in the HPIV-4 group (Table 1), whereas, sputum was accompanied by HPIV infection most frequently in the HPIV-4 group (*P* = 0.001, Table 1). Each virus type caused a range of illnesses, from FWLS to pneumonia (Table 1). Croup was diagnosed most frequently in the HPIV-1 group (*P* < 0.001), while none in the HPIV-4 group were diagnosed with croup (Table 1). About half of the patients in the HPIV-2 and HPIV-3 groups were diagnosed with URI, whereas ≥ 70% of the patients in the HPIV-1 and HPIV-4 groups were diagnosed with croup or LRI (Table 1). LRI developed most frequently in the HPIV-4 group (*p* = 0.002). Accordingly, abnormal breath sounds and chest x-ray abnormalities were more frequent in the HPIV-1 and HPIV-4 groups than in the HPIV-2 and HPIV-3 groups (*P* = 0.004, Table 1). However, severe complications were rare, and their frequencies were comparable among the four groups. There were no significant differences in the laboratory test results among the four groups.

**Table 1:** Comparison of the clinical characteristics of the pediatric patients between the four HPIV groups

Factor	HPIV-1 group (*N* = 49)	HPIV-2 group (*N* = 18)	HPIV-3 group (*N* = 84)	HPIV-4 group (*N* = 39)	*P* value
Male sex	28 (57.1)	11 (61.1)	47 (56.0)	28 (71.8)	0.390
Age, months	23 (11–32)	44 (21–62)	20 (10–30)	32 (14–66)	0.002
*Age group*					0.015
< 12 months	14 (28.6)	2 (11.1)	26 (31.0)	10 (25.6)	
12–23 months	12 (24.5)	3 (16.7)	27 (32.1)	8 (20.5)	
24–35 months	14 (28.6)	3 (16.7)	14 (16.7)	4 (10.3)	
≥ 36 months	9 (18.4)	10 (55.6)	17 (20.2)	17 (43.6)	
Underlying disease	4 (8.2)	2 (11.1)	6 (7.1)	8 (20.5)	0.142
*Symptoms*					
Fever	48 (98.0)	18 (100.0)	80 (95.2)	30 (76.9)	< 0.001
Fever days	4 (3–5)	2 (1–4)	4 (2–5)	2 (1–4)	0.005
Cough	48 (98.0)	15 (83.3)	73 (86.9)	35 (89.7)	0.155
Rhinorrhea	40 (81.6)	13 (72.2)	68 (81.0)	26 (66.7)	0.267
Sputum	39 (79.6)	7 (38.9)	54 (64.3)	33 (84.6)	0.001
Dyspnea	4 (8.2)	1 (5.6)	6 (7.1)	4 (10.3)	0.918
Vomiting	7 (14.3)	4 (22.2)	5 (6.0)	2 (5.1)	0.078
Diarrhea	5 (10.2)	3 (16.7)	6 (7.1)	1 (2.6)	0.278
Abdominal pain	4 (8.2)	3 (16.7)	2 (2.4)	1 (2.6)	0.058
Rash	1 (2.0)	0 (0.0)	2 (2.4)	2 (5.1)	0.677
Seizures	1 (2.0)	2 (11.1)	8 (9.5)	1 (2.6)	0.203
*Diagnosis*					
FWLS	0 (0.0)	2 (11.1)	5 (6.0)	0 (0.0)	0.061
URI	11 (22.4)	10 (55.6)	38 (45.2)	11 (28.2)	0.012
Croup	13 (26.5)	2 (11.1)	6 (7.1)	0 (0.0)	< 0.001
Bronchitis/bronchiolitis	25 (51.0)	4 (22.2)	35 (41.7)	24 (61.5)	0.029
Pneumonia	2 (4.1)	0 (0.0)	1 (1.2)	4 (10.3)	0.074
*Abnormal breath sounds*	30 (61.2)	3 (16.7)	31 (36.9)	19 (48.7)	0.004
Rales	10 (20.4)	1 (5.6)	14 (16.7)	10 (25.6)	0.304
Wheezing	5 (10.2)	1 (5.6)	11 (13.1)	10 (25.6)	0.111
Rhonchi	7 (14.3)	0 (0.0)	6 (7.1)	5 (12.8)	0.236
Stridor	10 (20.4)	1 (5.6)	5 (6.0)	0 (0.0)	0.004
Chest x-ray abnormalities	25 (51.0)	4 (22.2)	37 (44.0)	27 (69.2)	0.006
WBC count, /mm^3^	8800 (6400–11,600)	11,050(8700–13,400)	9550 (7550–12,600)	10,200 (8350–11,750)	0.285
Hemoglobin, g/dL	12.0 (11.5–12.8)	11.9 (11.1–12.9)	12.2 (11.4–12.8)	12.5 (11.8–13.0)	0.441
Platelet count, /mm^3^	250,000 (212,000–325,000)	246,500 (211,000–307,000)	278,000 (230,500–363,500)	303,000 (232,000–332,000)	0.247
ESR, mm/h	8 (2–18)	8 (4–12)	8 (2–17)	11 (5–21)	0.454
C-reactive protein, mg/dL	0.82 (0.20–1.41)	0.75 (0.14–2.15)	0.71 (0.25–2.28)	0.66 (0.19–2.20)	0.851
AST level elevation^a^	8 (16.3)	1 (5.6)	10 (11.9)	7 (17.9)	0.547
ALT level elevation^a^	3 (6.3)	0 (0.0)	6 (7.1)	3 (7.7)	0.699
LDH level elevation^a^	0 (0.0)	0 (0.0)	1 (1.2)	1 (2.6)	0.680
*Severe complications*					
Oxygen therapy	1 (2.0)	1 (5.6)	2 (2.4)	4 (10.3)	0.180
ICU admission	0 (0.0)	0 (0.0)	0 (0.0)	1 (2.6)	0.273

Data are presented as numbers (%) or median (interquartile range)

*HPIV* Human parainfluenza virus, *FWLS* Fever without localizing signs, *URI* Upper respiratory tract infection, *WBC* White blood cell, *ESR* Erythrocyte sedimentation rate, *AST* Aspartate transaminase, *ALT* Alanine transaminase, *LDH* Lactate dehydrogenase, *ICU* Intensive care unit

^**a**^AST, ALT, and LDH level elevations were defined when their serum levels exceeded the normal upper limits

During the COVID-19 pandemic in 2020–2021, 12 (80.0%) of 15 single HPIV infections were caused by HPIV-3. Significantly more patients in the HPIV-3 group were diagnosed with URI during the COVID-19 pandemic than before the COVID-19 pandemic (83.3% vs. 38.9%, *P* = 0.004, Table 2). Therefore, the frequencies of abnormal breath sounds (*P* = 0.048) and chest x-ray abnormalities (*P* = 0.007) decreased significantly during the COVID-19 pandemic compared with before the pandemic (Table 2). In addition, more patients experienced seizures during the COVID-19 pandemic than before the COVID-19 pandemic (50.0% vs. 2.8%, *P* < 0.001, Table 2).

**Table 2:** Comparison of the clinical characteristics of the HPIV-3 group between before the COVID-19 pandemic and during the COVID-19 pandemic

Factor	Before the COVID-19 pandemic (*N* = 72)	During the COVID-19 pandemic (*N* = 12)	*P* value
Male sex	40 (55.6)	7 (58.3)	0.858
Age, months	19 (10–29)	25 (10–33)	0.466
Underlying disease	6 (8.3)	0 (0.0)	0.587
*Symptoms*			
Fever	68 (94.4)	12 (100.0)	> 0.99
Cough	64 (88.9)	9 (75.0)	0.188
Rhinorrhea	61 (84.7)	7 (58.3)	0.046
Sputum	50 (69.4)	4 (33.3)	0.023
Dyspnea	6 (8.3)	0 (0.0)	0.587
Vomiting	4 (5.6)	1 (8.3)	0.547
Diarrhea	6 (8.3)	0 (0.0)	0.587
Abdominal pain	2 (2.8)	0 (0.0)	> 0.99
Rash	2 (2.8)	0 (0.0)	> 0.99
Seizures	2 (2.8)	6 (50.0)	< 0.001
*Diagnosis*			
FWLS	4 (5.6)	1 (8.3)	0.547
URI	28 (38.9)	10 (83.3)	0.004
Croup	6 (8.3)	0 (0.0)	0.587
Bronchitis/bronchiolitis	34 (47.2)	1 (8.3)	0.011
Pneumonia	1 (1.4)	0 (0.0)	> 0.99
*Abnormal breath sounds*	30 (41.7)	1 (8.3)	0.048
Rales	13 (18.1)	1 (8.3)	0.681
Wheezing	11 (15.3)	0 (0.0)	0.351
Rhonchi	6 (8.3)	0 (0.0)	0.587
Stridor	5 (6.9)	0 (0.0)	> 0.99
Chest x-ray abnormalities	36 (50.0)	1 (8.3)	0.007
WBC count, /mm^3^	10,250 (7550–12,750)	9000 (7550–11,050)	0.565
Hemoglobin, g/dL	12.2 (11.4–12.8)	12.1 (10.9–12.7)	0.735
Platelet count, /mm^3^	278,000 (221,500–362,500)	273,500 (247,000–366,000)	0.749
ESR, mm/h	10 (4–20)	2 (2–3)	< 0.001
C-reactive protein, mg/dL	0.79 (0.28–2.28)	0.28 (0.09–1.85)	0.140
AST level elevation^a^	8 (11.1)	2 (16.7)	0.630
ALT level elevation^a^	5 (6.9)	1 (8.3)	> 0.99
LDH level elevation^a^	1 (1.4)	0 (0.0)	> 0.99
*Severe complications*			
Oxygen therapy	2 (2.8)	0 (0.0)	> 0.99
ICU admission	0 (0.0)	0 (0.0)	NA

Data are presented as numbers (%) or median (interquartile range)

*HPIV* Human parainfluenza virus, *COVID-19* Coronavirus disease 2019, *FWLS* Fever without localizing signs, *URI* Upper respiratory tract infection, *WBC* White blood cell, *ESR* Erythrocyte sedimentation rate, *AST* Aspartate transaminase, *ALT* Alanine transaminase, *LDH* Lactate dehydrogenase, *ICU* Intensive care unit, *NA* Not available

^**a**^AST, ALT, and LDH level elevations were defined when their serum levels exceeded the normal upper limits

## Discussion

The seasonal distributions and clinical characteristics of HPIV infection were determined by serotype in this study. HPIV-3 showed a peak in spring, while the other three serotypes showed peaks in autumn. There were some differences in the clinical characteristics among the four HPIV serotypes and over time.

In this study, it was reaffirmed that HPIV infection occurred mostly in young children, with a peak between spring and autumn, and caused a range of respiratory tract infections [1, 3, 8, 10]. HPIV-3 was the most prevalent among the four serotypes, consistent to the previously reported results [10, 11, 15, 17–19], and similar seasonal trends and clinical characteristics of HPIV-3 infection to those previously reported were identified: HPIV-3 was prevalent in spring and summer annually [1, 3, 8–11, 15, 17–19], patients infected by HPIV-3 tended to be younger than those infected by other serotypes [1, 3, 9, 18], and croup was diagnosed infrequently [3, 8, 9, 16–18]. Therefore, we can regard HPIV infections in spring and summer as non-specific URI or LRI rather than croup, caused by HPIV-3 in young children and infants.

Serotypes other than HPIV-3 showed different seasonality. Most previous epidemiological studies showed biennial epidemics of HPIV-1 and HPIV-2 in autumn [1, 3, 10, 11]. In this study, HPIV-1 was epidemic in annual autumn in 2015–2017, consistent with previously reported results in Korean children in 2013–2017 [17, 18]. Whereas, HPIV-1 infection was widespread from spring to autumn in 2018 and 2019, which was consistent with a Chinese study showing fluctuations without distinct seasonality of HPIV-1 [15, 21]. The seasonality of HPIV-2 was more varied than that of HPIV-1: epidemics in biennial autumn, annual autumn, and annual winter were reported [1, 3, 9–11, 17, 18, 22], and some studies failed to find a distinct seasonality of HPIV-2 [8, 15, 16, 19, 21]. In this study, HPIV-2 was prevalent in autumn as a whole; however, a regular seasonality could not be defined due to the lowest number of HPIV-2 identification among the four serotypes, clustered in 2015 and 2019. HPIV-4 was not tested or was identified least frequently among the four serotypes before the introduction of PCR tests [3, 8, 9, 22], and therefore, information on its seasonality and clinical characteristics was insufficient. With the use of PCR tests, HPIV-4 infection has been identified more frequently than HPIV-2 infection [15–18, 21], and therefore, its clinical significance was required to be determined. In this study, HPIV-4 was prevalent in autumn as a whole; however, it was prevalent in spring in 2017 and the numbers of identification varied year to year. Nationwide studies for several years in the UK and USA reported annual epidemics of HPIV-4 in autumn [10, 11], while Korean and Chinese studies with ≤ 5 years of study period reported fluctuating seasonal patterns of HPIV-4 [15, 17–19, 21]. As a result, the seasonality of HPIV types 1, 2, and 4 varied according to geographic area, study duration, patient number, and identification method in previous reports and this study. Moreover, seasonal epidemiology tended to be different over time even in a same country. Although HPIV-3 was most prevalent with an annual epidemic in spring consistently in temperate areas, HPIV-3 did not exhibit a distinct seasonality or other serotypes were more predominant than HPIV-3 in tropical and subtropical areas [23, 24]: a climate factor cannot be ignored. Therefore, seasonal epidemiology of HPIV infections should be determined in each country independently by observing for sufficient years, and its changing trends should be traced.

Considering that HPIV-2 infections were least frequent among serotypes prevalent in autumn and less than a quarter of them manifested as LRI, clinical differentiation between HPIV-1 and HPIV-4 infections might be emphasized in patients infected by HPIV in autumn. Except that more patients in the HPIV-1 group were aged < 36 months, febrile, and diagnosed with croup than those in the HPIV-4 group, similar clinical, radiological, and laboratory findings and outcomes were identified between HPIV-1 and HPIV-4 groups in this study. Therefore, clinical differentiation between HPIV-1 and HPIV-4 infections seems to have little clinical impact. We can only consider that LRIs in young children and most cases of croup are caused by HPIV-1, and LRIs in old children are caused by HPIV-4 in autumn.

During the COVID-19 pandemic, respiratory and gastrointestinal infectious diseases decreased with intensive infection control and prevention strategies in Korea [25]. Accordingly, the numbers of HPIV identified in 2020–2021 were significantly lower than those in 2015–2019. Moreover, different seasonality and clinical manifestations of HPIV infections from those before the COVID-19 pandemic were observed in 2021: HPIV-3 was prevalent in autumn, not in spring, significantly more patients were diagnosed with URI and experienced seizures in the HPIV-3 group than before. Before the COVID-19 pandemic, seizures occurred in 2.8% of the HPIV-3 group and were accompanied by HPIV-2 infection most frequently (11.1%). Previously, HPIV was identified in about 10% of children with febrile seizures [1, 26], and 9–16% of pediatric patients with HPIV infections experienced seizures [1, 27]. Due to the small number of patients during the COVID-19 pandemic, we could not evaluate why the seasonality and clinical characteristics of HPIV infection changed. A new genetic lineage of HPIV-3 circulated in Korea in 2021 was identified, and multiple HN gene mutations were found by genetic analysis of the HN gene [28]. However, the clinical significance of these genetic variations could not be defined [28]. The resurgence of HPIV infection in 2021 was also observed in China and Japan, neighboring countries of Korea, and in western countries with the relaxation of social knockdown [29–34]. Because immunity to HPIV infection is transient and wanes over time [4], the absence of re-infections in the community during social knockdown could increase the population susceptible to HPIV infection [34]. Considering that HPIV-3 tends to infect younger children than other HPIV serotypes, the exemption of mask use for children aged < 24 months could be associated with the resurgence of HPIV-3 infection, as was observed with the resurgence of RSV infection in 2021 in several countries including Korea [30]. Moreover, the mass migration of the population during the Korean Thanksgiving in late September might increase the transmission of HPIV in the community in Korea. Further follow-up studies should be conducted after the cessation of the COVID-19 pandemic to trace the trends of seasonal epidemiology and clinical characteristics of HPIV infections.

This study had some limitations including a selection bias due to its retrospective nature. Especially, this study included inpatients only, and therefore, outpatients with mild symptoms were not included and disease severity might be overestimated. Previous studies including outpatients reported the frequency of LRI as < 10% [3, 8]. However, a similar seasonality of HPIV between a community surveillance cohort and hospitalized patients was reported [35]. Despite seven years of study duration, a small number of patients were included during the last two years due to the COVID-19 pandemic in this study. Considering geographic and climate factors on the seasonality of HPIV, the results of this study might not be applicable to other countries; however, they are worth to note in temperate countries. Genetic analyses for HPIV were not performed in this study. Compared with RSV, which belongs to the *Paramyxoviridae* family, information on genetic variants and molecular epidemiology is more limited for HPIV. Further multinational studies including many patients over several years are needed to define the genotypes of HPIV and to evaluate the epidemiological and clinical characteristics of each HPIV genotype.

## Conclusions

In conclusion, in Korea with a temperate climate, HPIV-3 was prevalent in spring, and the other serotypes were prevalent in autumn. Although the differences of clinical characteristics among three prevalent serotypes in autumn seemed to have insignificant impact, HPIV-4 acted as a significant lower respiratory pathogen. The seasonality of HPIV varied according to geographic area, climate, study duration, patient characteristics, and laboratory test method, and the COVID-19 pandemic might influence the seasonality and clinical manifestations of HPIV infections. Follow-up studies including pre- and post-COVID-19 periods of sufficient years should be performed independently in each geographic area.

## Data Availability

The datasets used and analyzed during the current study are available from the corresponding author on reasonable request.

## Abbreviations

COVID-19: Coronavirus disease 2019
FWLS: Fever without localizing signs
HN: Hemagglutinin-neuraminidase
HPIV: Human parainfluenza virus
LRI: Lower respiratory tract infection
PCR: Polymerase chain reaction
RSV: Respiratory syncytial virus
URI: Upper respiratory tract infection
